# Retraction Note: Inhibition of EZH2 enhances the therapeutic effect of 5-FU via PUMA upregulation in colorectal cancer

**DOI:** 10.1038/s41419-023-05764-6

**Published:** 2023-03-29

**Authors:** Xiao Tan, Zhongqiang Zhang, Ping Liu, Hongliang Yao, Liangfang Shen, Jing-Shan Tong

**Affiliations:** 1 Department of Oncology, Xiangya Hospital, Central South University, Changsha, Hunan Province 410008 People’s Republic of China; 2 Department of Liver Transplantation, The Second Xiangya Hospital of Central South University, Changsha, Hunan Province 410011 People’s Republic of China; 3 Department of General Surgery, The Second Xiangya Hospital of Central South University, Changsha, Hunan Province 410011 People’s Republic of China; 4 Department of Pharmacology and Chemical Biology, University of Pittsburgh School of Medicine, Pittsburgh, PA 15213 USA

**Keywords:** Chemotherapy, Colon cancer

Retraction to: *Cell Death and Disease* 10.1038/s41419-020-03266-3, published online 12 December 2020

The Editors-in-Chief have retracted this article at the authors’ request. After publication, concerns were raised regarding image similarities between this article and a number of previously published articles, specifically:

Several images in Fig. 6D and E appear highly similar to those in Fig. 6C and D in [1], Fig. 6D and E in [2], Fig. 5E and F in [3], and Fig. 7E in [4].

Fig. 5E input appears highly similar to Fig. 4E input in [1].

The authors have stated that the images in Fig. 6 were misused and could not be reproduced, which undermined the reliability of the conclusions. The Editors-in-Chief therefore no longer have confidence in the presented data and the conclusions of the article.

All authors agree to this retraction.
